# A Novel Protein Recovery Method via Gel Separation and Silica Columns Capable of Rapid Antigen Purification and Deep Proteomic Identification

**DOI:** 10.3390/molecules31183307

**Published:** 2026-09-17

**Authors:** Dan Wang, Jianhong Wu, Xingmei Zheng, Ziquan Fan, Zhexuan Li, Cunzhi Peng, Bingqiang Xu, Zheng Tong

**Affiliations:** 1 Key Laboratory of Biology and Genetic Resources of Tropical Crops, Institute of Tropical Bioscience and Biotechnology, Chinese Academy of Tropical Agricultural Sciences & Hainan Institute for Tropical Agricultural Resources, Haikou 571101, China; 2 Thermo Fisher Scientific (China) Co., Ltd., 2517 Jinke Road, Pudong New Area, Shanghai 201206, China; 3 State Key Laboratory of Tropical Crop Breeding, Institute of Tropical Bioscience and Biotechnology, Chinese Academy of Tropical Agricultural Sciences, Sanya 572024, China

**Keywords:** gel separation-based protein recovery method, silica column, protein purification, antigen purification, molecular mass-based proteomic pre-fractionation, proteomic identification

## Abstract

Gel electrophoresis is widely used for protein and nucleic acid analysis. Nucleic acid recovery from agarose gels is convenient and efficient, but protein recovery is not. This study aimed to develop and validate a novel method for recovering proteins from gels. Methods: After protein separation on gels, excised gel slices containing target proteins are processed using the same silica-column workflow as that used for nucleic acid purification, but with buffers specifically adapted for protein recovery. This approach only requires brief centrifugation steps and avoids laborious extraction and specialized equipment. Results: Proteome-wide recovery showed a slight preference of the method for low-molecular-mass and acidic proteins, while individual protein recovery efficiencies ranged from 69.9% to 86.7% and showed no correlation with intrinsic protein properties. The method effectively supported antigen purification. A banana (*Musa acuminata* AAA group ‘Brazilian’) proteome library constructed from recovered fractions contained over 18,000 protein groups and 27,000 individual proteins, which significantly enhanced protein identification in library-based data-independent acquisition (DIA) compared to directDIA analysis of single samples. Conclusions: This method bridges gel-based protein separation- and column-based purification, making protein purification as simple, fast, and scalable as nucleic acid purification. It offers a practical alternative for proteomic sample preparation, antigen purification, and DIA workflows.

## 1. Introduction

Protein separation and purification represent fundamental techniques in biological research. Since the 1980s, the groundbreaking development of fast protein liquid chromatography (FPLC) [1] has driven substantial progress in protein purification, establishing chromatographic methods as the gold standard for therapeutic protein manufacturing [2]. Integrated strategies combining tag-mediated affinity chromatography, size-exclusion chromatography, ion-exchange chromatography, reversed-phase chromatography, and immobilized metal affinity chromatography are widely employed in applications ranging from antibody production to structural characterization and therapeutic screening [3,4]. The superior throughput, resolution, and automation of these chromatographic systems have cemented their dominance in industrial and biomedical protein purification workflows [4]. However, high consumable costs, time-intensive procedures, and the need for target-specific optimization remain major limitations to their broader application.

Beyond chromatography, gel electrophoresis serves as another mainstream protein separation methodology, with SDS-polyacrylamide gel electrophoresis (SDS-PAGE) being the most widely used approach [5]. For protein recovery from polyacrylamide gels (PAG), the conventional protocol involves excising target bands followed by mechanical disruption and buffer extraction [6,7]. This method gained prominence during the MALDI-MS era, particularly for in-gel digestion and identification of protein spots from two-dimensional electrophoresis (2D-PAGE) [8,9]. Electroelution represents an alternative purification approach, achieving significant reductions in processing time and improved recovery through continuously evolving modular devices [10,11,12,13]. Nevertheless, its widespread adoption is hindered by the requirement for specialized instrumentation and necessary downstream purification and concentration steps. To further purify polyacrylamide gel (PAG) extractions, multi-stage strategies such as methanol-chloroform-water precipitation [14], anion-exchange solid-phase extraction [15], and carboxylate-modified magnetic bead adsorption [16] have been developed.

Building on recent advances in high-concentration agarose gel (HAG, 5–14% (*m*/*v*)) preparation, which enables high-resolution separation of complex protein mixtures [17], we present an innovative protein purification strategy based on HAG separation and dissolution. Previous studies have utilized matrices such as StrataClean resin to adsorb proteins from chaotropic salt-dissociated agarose gels [18], or carboxylate-modified magnetic beads to purify proteins from PAG eluates [16]. In this study, we planned to develop a HAG separation-based protein purification method using low-cost and widely available silica columns. By designing a dedicated reagent system (binding/rinsing/elution) optimized for protein binding and retaining an operational workflow similar to nucleic acid purification protocols, this approach could enable rapid target protein purification.

## 2. Results

### 2.1. Buffer Selection for Silica Column-Based Protein Recovery

**Figure 1:**
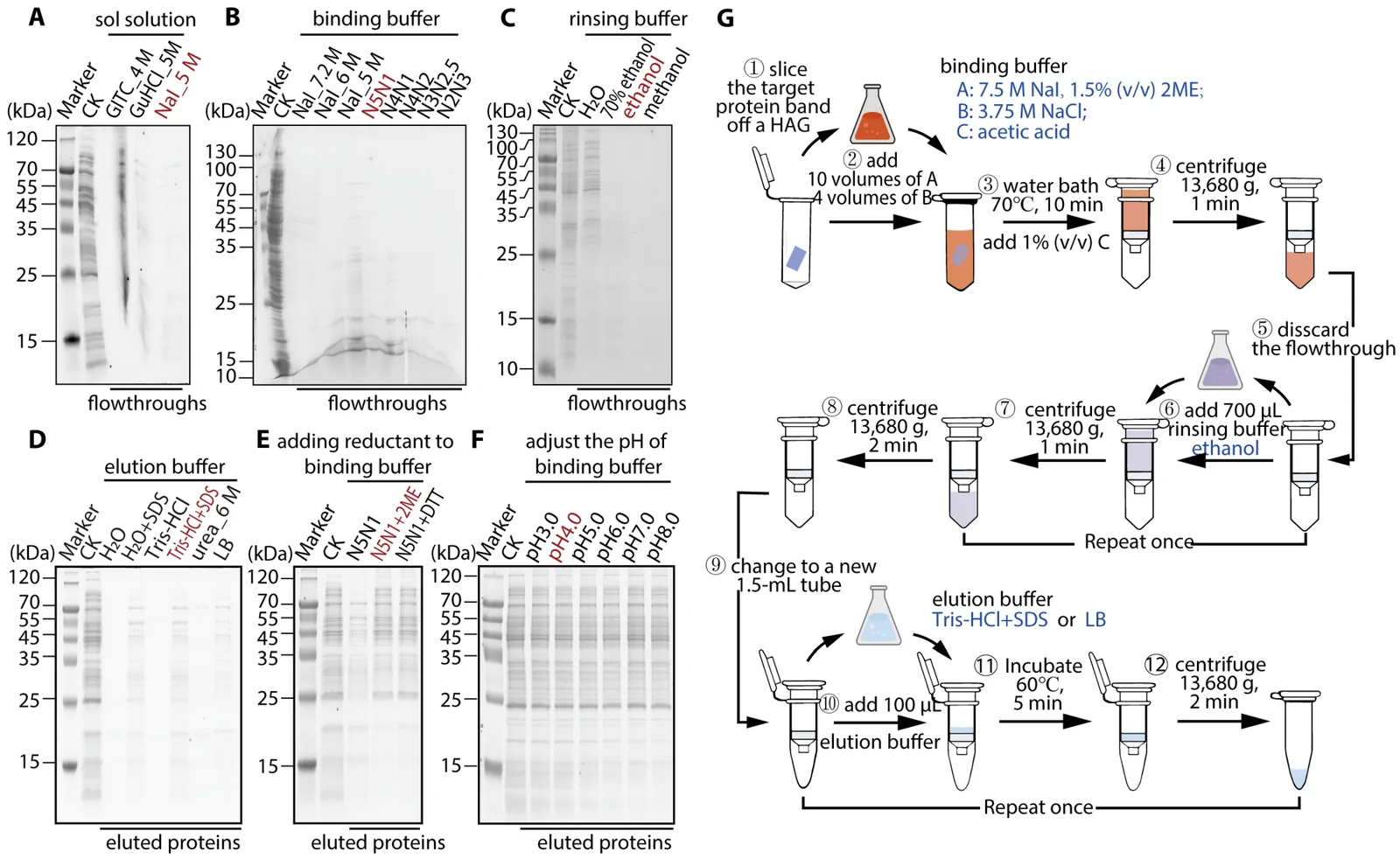
Development of a silica column-based protein recovery method. (**A**) Screening of chaotropic salts for the gel-dissolving buffer. (**B**) Optimization of the binding buffer composition. (**C**) Screening of rinsing buffers. (**D**) Screening of the elution buffers. (**E**) Effect of reducing agents in the binding buffer on protein recovery. (**F**) Influence of binding buffer pH on recovery efficiency. (**G**) Schematic workflow of the protein recovery procedure. Marker, prestained color protein ladder (10–170 kDa, Beyotime Biotechnology, Shanghai, China), CK, 25 μg or 50 μg (only panel (**B**)) of *Escherichia coli* proteins prior to recovery; recovered proteins or flow-throughs were loaded in the same proportion as the pre-recovery input. GITC_4M, 4 M guanidine isothiocyanate; GuHCl_5M, 5 M guanidine hydrochloride; NaI_5M, 5 M sodium iodide; NaI_7.2 M, 7.2 M NaI; NaI_6 M, 6 M NaI; N5N1, 5 M NaI and 1 M NaCl; N4N1, 4 M NaI and 1 M NaCl; N4N2, 4 M NaI and 2 M NaCl; N3N2.5, 3 M NaI and 2.5 M NaCl; N2N3, 2 M NaI and 3 M NaCl; H_2_O+SDS, 0.1% (*m*/*v*) SDS; Tris-HCl, Tris-HCl buffer (20 mM, pH 8.0); Tris-HCl+SDS, Tris-HCl buffer (20 mM, pH 8.0), 0.1% (*m*/*v*) SDS; urea_6 M, 6 M urea; LB, lysis buffer (7 M urea, 2 M thiourea, 4% (*m*/*v*) CHAPS, 2% (*m*/*v*) DTT, pH 8.5); N5N1+2ME, 5 M NaI, 1 M NaCl and 1% (*v*/*v*) β-mercaptoethanol; N5N1+DTT, 5 M NaI, 1 M NaCl and 1% (*v*/*v*) Dithiothreitol.

During the development of the silica column-based protein recovery method, an initial screening of suitable protein binding buffers was conducted. Three high-concentration chaotropic salts were evaluated for gel dissolution, with the final solution containing 4 M guanidine thiocyanate (GITC), 5 M guanidine hydrochloride (GuHCl), or 5 M NaI. All three effectively and rapidly dissolved high-concentration agarose gels (5–10% (*m*/*v*) HAG). However, the flow-through from the NaI-containing buffer showed the lowest protein content (Figure 1A), suggesting that NaI alone could serve a dual function in both gel dissolution and protein binding. Increasing the NaI concentration or supplementing with NaCl in the binding buffer further reduced protein loss in the flow-through (Figure 1B), thereby improving the column’s protein-binding capacity. Balancing gel dissolution efficiency and binding performance, we determined that the binding buffer should contain at least 5 M NaI and 1 M NaCl in the final gel-dissolution solution.

Among H_2_O, 70% (*v*/*v*) ethanol, anhydrous ethanol, and methanol tested as rinsing buffers, no significant protein traces were detected in the flow-through from anhydrous ethanol or methanol (Figure 1C). Consequently, absolute ethanol was selected as the rinsing buffer. During elution, both SDS solution and lysis buffer (LB) containing urea and thiourea—but not H_2_O or Tris-HCl buffer (20 mM, pH 8.0)—effectively eluted proteins from the columns (Figure 1D). Although SDS buffer showed higher elution efficiency, LB offered the advantage of maintaining an SDS-free environment (Figure S1). Nevertheless, overall protein recovery efficiency remained suboptimal (Figure 1D). Minor losses of low-molecular-mass (LM_r_) proteins were observed in the flow-through of binding buffers (Figure 1B,C), indicating that poor adsorption might contribute to incomplete LM_r_ protein recovery. However, the generally low recovery appeared to result primarily from proteins remaining tightly bound to the silica membrane and resisting elution.

During subsequent optimization, we observed that incorporating the reducing agents β-mercaptoethanol (2ME) or DL-Dithiothreitol (DTT) into the binding buffer substantially enhanced overall protein recovery efficiency (Figure 1E). Since the addition of 2ME did not reduce protein content in the flow-through but significantly increased the final recovery yield (Figure S2), we inferred that the reducing agent improves purification performance not by enhancing protein binding to the silica membrane, but likely by promoting more effective protein elution. Although the reducing agents did not fully resolve the issue of LM_r_ protein recovery, further adjustments to the binding buffer pH were investigated. A modest improvement in the recovery of LMr proteins was observed upon reduction in the pH to below 5 (Figure 1F).

We also evaluated protein recovery performance in solubilized gel solutions with varying agarose concentrations (Figure S3). Excessively high agarose concentrations moderately reduced recovery efficiency, likely due to impaired contact and adsorption between proteins and the silica membrane. Conversely, lower agarose concentrations increased solution volume and required more frequent column passages, prolonging experimental duration and elevating the risk of protein loss. Therefore, an optimal final agarose concentration of approximately 0.6% (*m*/*v*) was determined (Figure S3), typically achieved by adding 10 volumes of 7.5 M NaI and 4 volumes of 3.75 M NaCl per volume of gel piece. Based on these optimized buffer conditions, a complete protein recovery workflow was established (Figure 1G).

### 2.2. Silica Columns Selection

**Figure 2:**
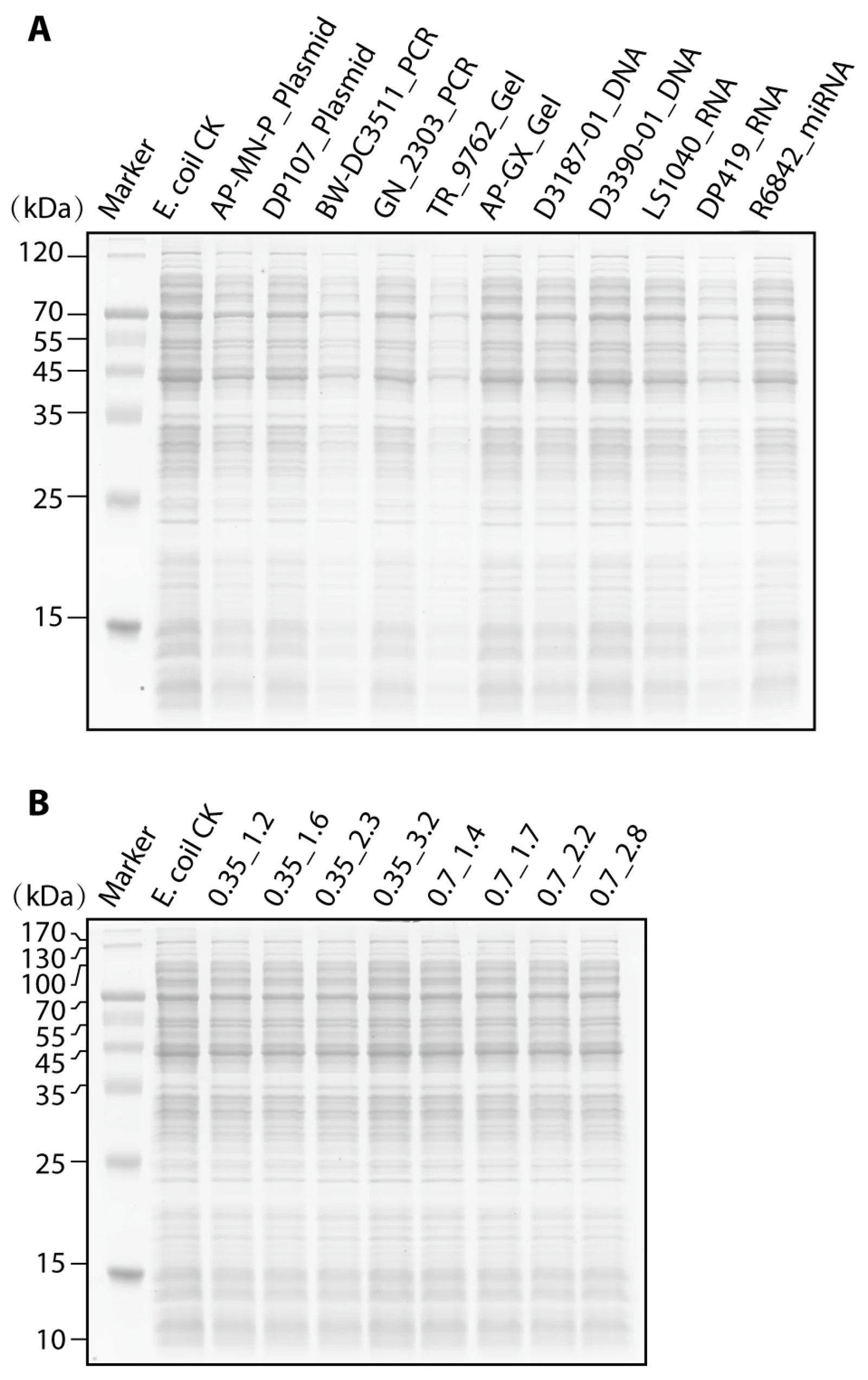
Protein recovery using different silica columns. (**A**) Recovery of total *E. coli* protein using silica columns from different nucleic acid purification kits. (**B**) Recovery of total *E. coli* protein using silica columns with varying pore sizes from the same manufacturer. Proteins recovered from 200 μg of *E. coli* protein were loaded at 1/8 of the eluent volume; CK, 25 μg of protein before recovery. 0.35, 0.35 μm pore size silica column; 0.7, 0.7 μm pore size silica column; 1.2, 1.6, 2.3, 3.2, 1.4, 1.7, 2.2, 2.8, the thickness (in mm) of the silica membrane in each column. Silica column sources are detailed in Table S1.

Theoretically, protein recovery efficiency is influenced not only by buffer composition but also by silica column characteristics. During method development, we initially utilized silica columns sourced from the AxyPrep™ plasmid miniprep kit (Corning Incorporated, Corning, NY, USA) and the Eastep™ super total RNA extraction kit (Promega Corp, Madison, WI, USA). Subsequently, we tested silica columns from various commercial kits designed for PCR product purification, DNA gel recovery, plasmid extraction, DNA extraction, and RNA extraction kits. We observed variations in the recovery efficiency of 200 μg *E. coli* protein across different silica columns, although no clear correlation with kit type was identified (Figure 2A). The unspecified specifications of the silica columns in these kits made it difficult to determine which column characteristics are more suitable for protein recovery.

Based on the principles of nucleic acid recovery, large-pore silica columns are typically used for high-molecular-mass nucleic acids (e.g., genomic DNA and plasmids), while small-pore, highly hydroxylated silica columns enhance the binding efficiency of low-molecular-mass nucleic acids (e.g., cell-free DNA and MicroRNA). Given that proteins generally have lower molecular mass compared to DNAs, small-pore silica columns may be more appropriate for protein recovery. However, when we compared the protein recovery performance between bulk silica columns with 0.35 μm and 0.7 μm pore-sized membranes from the same manufacturer, no significant difference was observed. Similarly, the thickness of the silica membrane showed no notable effect on recovery efficiency (Figure 2B).

### 2.3. Preference Analysis of the Newly Developed Protein Recovery Method

**Figure 3:**
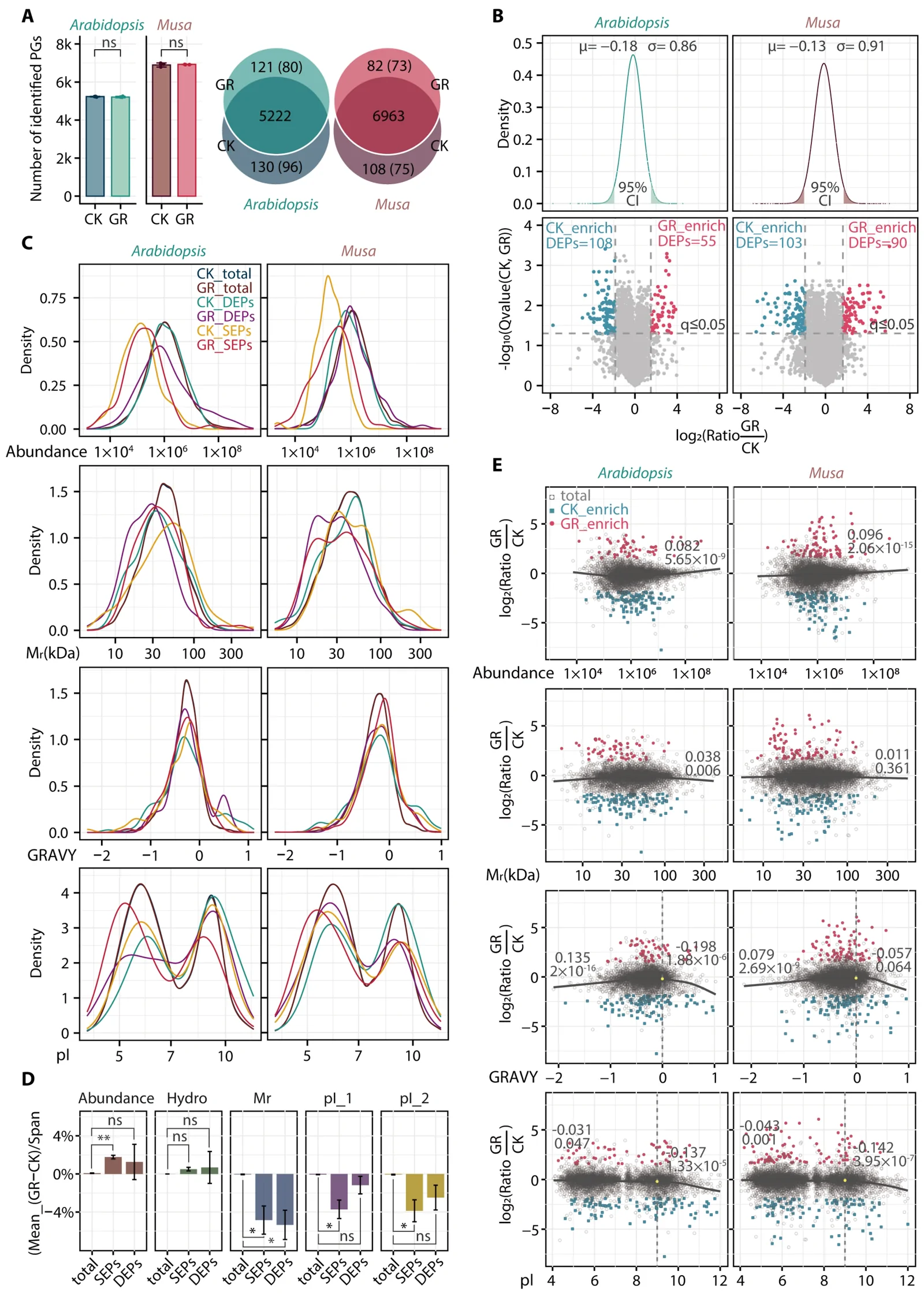
Preference analysis of the newly developed protein recovery method. (**A**) Number of protein groups identified in *Arabidopsis* and *Musa* pre-recovery (CK) and post-recovery (GR). PGs, protein groups. (**B**) Analysis of differentially expressed proteins (DEPs) between CK and GR samples. Normal distribution parameters (μ, σ) are indicated above each plot. The area between vertical dashed lines represents the 95% confidence interval (CI). (**C**) Density distributions of relative abundance (Abundance), theoretical molecular mass (M_r_), grand average of hydropathicity (GRAVY), and isoelectric point (pI) for proteins in CK and GR recovery. These include total proteins in CK (CK_total) and GR (GR_total); DEPs with increased abundance in CK (CK_DEPs) and increased abundance in GR (GR_DEPs); and specifically expressed proteins (SEPs) present only in CK (CK_SEPs) or GR (GR_SEPs). (**D**) Peak center shifts of the distribution curves of Abundance, M_r_, GRAVY, and pI for total proteins, SEPs, and enriched DEPs between CK and GR. pI_1 and pI_2 denote left and right peak center shifts, respectively, in bimodal Gaussian fittings of pI distribution. (**E**) Correlation between expression changes after recovery and protein abundance, M_r_, GRAVY, or pI. Spearman’s rank correlation was used; analyses against GRAVY and pI were segmented at GRAVY = 0 and pI = 9, respectively. Spearman’s correlation coefficients (ρ) and corresponding *p*-values are annotated above each panel. Student’s *t*-test was used in (**A**,**D**); *, *p* < 0.05, **, *p* < 0.01; ns, not significant.

To evaluate potential preference of the newly developed silica column-based protein recovery method toward specific protein properties, we performed mass spectrometry (MS) identification and comparative proteomic analysis on pre- and post-recovery samples of *Arabidopsis thaliana* (*Arabidopsis*) and *Musa acuminata* AAA group ‘Brazilian’ (*Musa*) proteome (Figure S4). The results demonstrated minimal differences in the total number of identified proteins between pre- and post-recovery samples (Figure 3A), with limited specific expressed proteins (SEPs) (Figure 3A) and differentially expressed proteins (DEPs) (Figure 3B) observed.

Analysis of the density distributions of protein abundance, molecular mass (M_r_), hydrophobicity, and isoelectric point (pI) across the whole proteome in both pre- and post-recovery samples revealed no significant shifts in these properties following recovery (Figure 3C), indicating that the newly established protein recovery method exhibits no substantial bias toward specific protein characteristics. However, subsequent analysis of SEPs and DEPs identified discernible alterations in their abundance, M_r_, and pI distributions. In both *Arabidopsis* and *Musa*, the abundance distributions of SEPs shifted toward the low-abundance range in both pre- and post-recovery samples (Figure 3C), a trend likely resulting from the limited MS detection sensitivity for low-abundance proteins. The grand average of hydropathicity (GRAVY) showed no obvious changes in either SEPs or DEPs. Notable shifts toward lower molecular mass were observed among SEPs and abundance-increased DEPs in post-recovery samples of both *Arabidopsis* and *Musa*. Furthermore, obviously acidic shifts in pI occurred in SEPs from post-recovery samples of both species (Figure 3C).

Normal distribution curves were fitted for protein abundance, M_r_, and GRAVY, while bimodal Gaussian curves were applied to pI distribution for total proteins, SEPs, and DEPs in both pre- and post-recovery samples (Table S2 and Figure S5). The whole proteome showed minimal center shifts (≤0.1%) across all properties after recovery. In contrast, center shifts for SEPs and DEPs ranged around 1–5%. Among these, the low M_r_ shift in post-recovery increased both SEPs and DEPs reached statistical significance compared to that of the whole proteome (Figure 3D), suggesting a potential methodological preference toward low M_r_ proteins.

Spearman correlation analysis between changes in protein abundance after recovery and intrinsic protein properties revealed significant correlations with protein abundance, M_r_, GRAVY, and pI. However, most correlations were weak, falling below conventional significance thresholds (|ρ| < 0.1) (Figure 3E). Given the large sample size characteristic of whole-proteome analyses, these weak associations hold negligible practical explanatory power, indicating negligible effect size (ρ^2^ < 0.01). Nonetheless, consistent weak yet significant negative correlations (0.1 < |ρ| < 0.3, *p* < 10^−4^) were observed between recovery-induced abundance changes and pI values above 9 in both the *Arabidopsis* and *Musa* proteomes, suggesting a potential methodological preference toward acidic proteins.

### 2.4. Recovery Efficiencies of Individual Proteins with Different Properties

**Figure 4:**
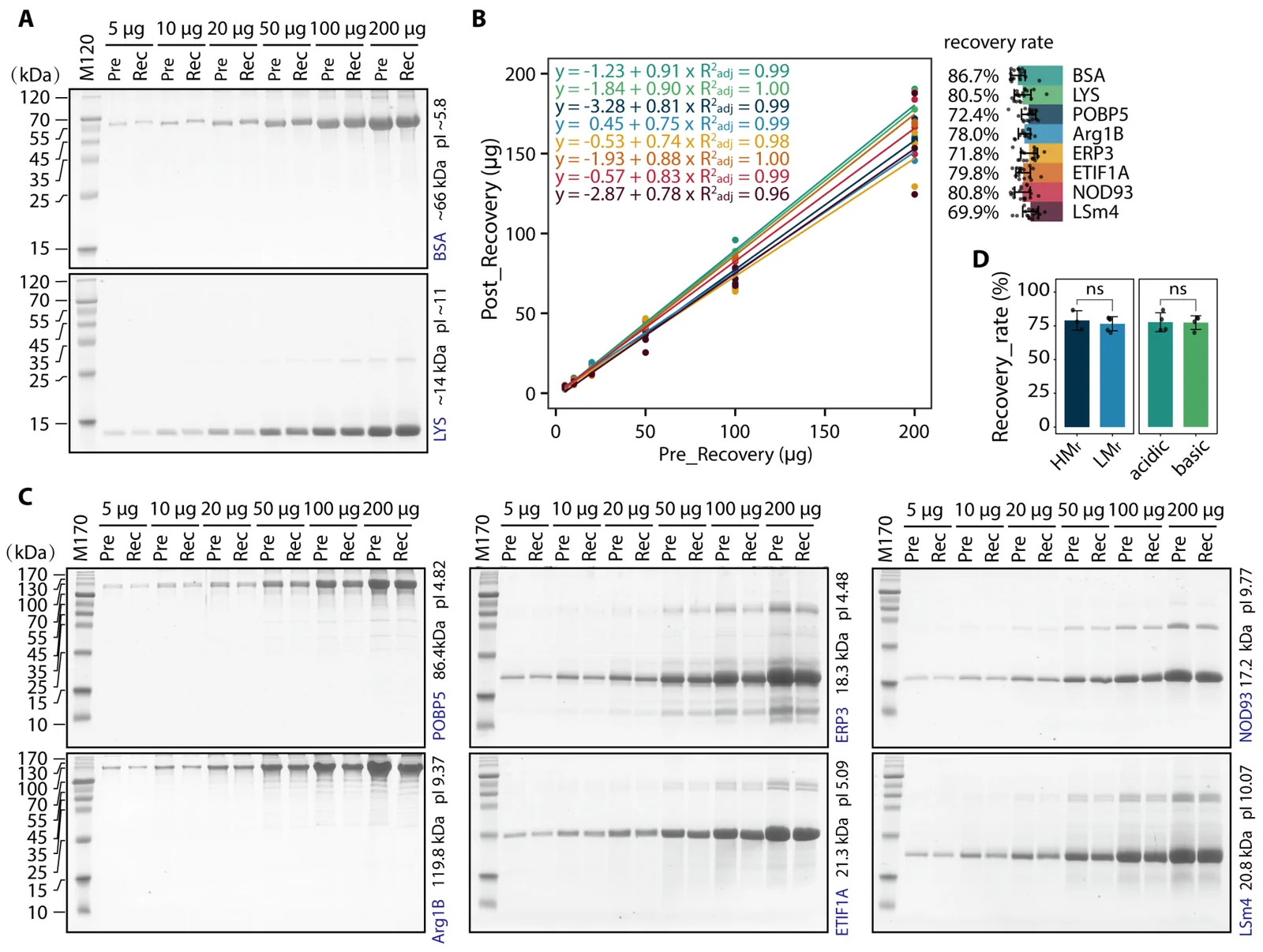
Analysis of protein recovery efficiency across protein properties. (**A**) Recovery of acidic bovine serum albumin (BSA) and basic lysozyme (LYS). (**B**) Linear regression analysis of recovery efficiency across varying protein properties. (**C**) Recovery of *Musa* proteins with distinct molecular mass (M_r_) and isoelectric point (pI). (**D**) Statistical comparison of the recovery efficiency between high-M_r_ (HM_r_) and low-M_r_ proteins (LM_r_), and between high-pI (basic) and low-pI (acidic) proteins. Each Lane was loaded with 1/8 of the pre-recovery protein input (Pre) or 1/8 of the post-recovered protein output (Rec). Statistical testing was performed using Student’s *t*-test; ns, not significant.

To characterize the protein recovery efficiencies across varying M_r_ and pI, we initially evaluated two commercial proteins, bovine serum albumin (BSA, ~66 kDa, pI ~4.7–5.8) and lysozyme (LYS, ~14 kDa, pI ~11), and observed the linear regressions ranging from 5 to 200 μg, with a linear coefficient of over 80% (Figure 4A,B). The LM_r_ and basic protein LYS exhibited a slightly lower recovery rate than the higher M_r_ acidic BSA.

To further explore this trend, we selected six additional proteins from *Musa* for prokaryotic expression and recovery assessment (Figure 4C). These included: the high-molecular-mass (HM_r_) acidic protein peroxisome biogenesis protein 5 (POBP5); the HM_r_ basic protein argonaute 1B-like (Arg1B); the LM_r_ acidic proteins elicitor-responsive protein 3-like (ERP3) and eukaryotic translation initiation factor 1A-like (ETIF1A); and the LM_r_ basic proteins early nodulin-93-like (NOD93) and probable U6 snRNA-associated Sm-like protein LSm4 (LSm4). As summarized in Figure 4B, all selected proteins exhibited recovery efficiencies between 69.9% and 86.7%. Student’s *t*-test analysis indicated no statistically significant differences in recovery efficiency between acidic (pI < 6) and basic (pI > 9) proteins, or between HM_r_ (M_r_ > 65 kDa) and LM_r_ (M_r_ < 25 kDa) proteins (Figure 4D). These results indicate that the current recovery method exhibits negligible variation across diverse protein properties.

### 2.5. Antigen Purification and Antibody Production

**Figure 5:**
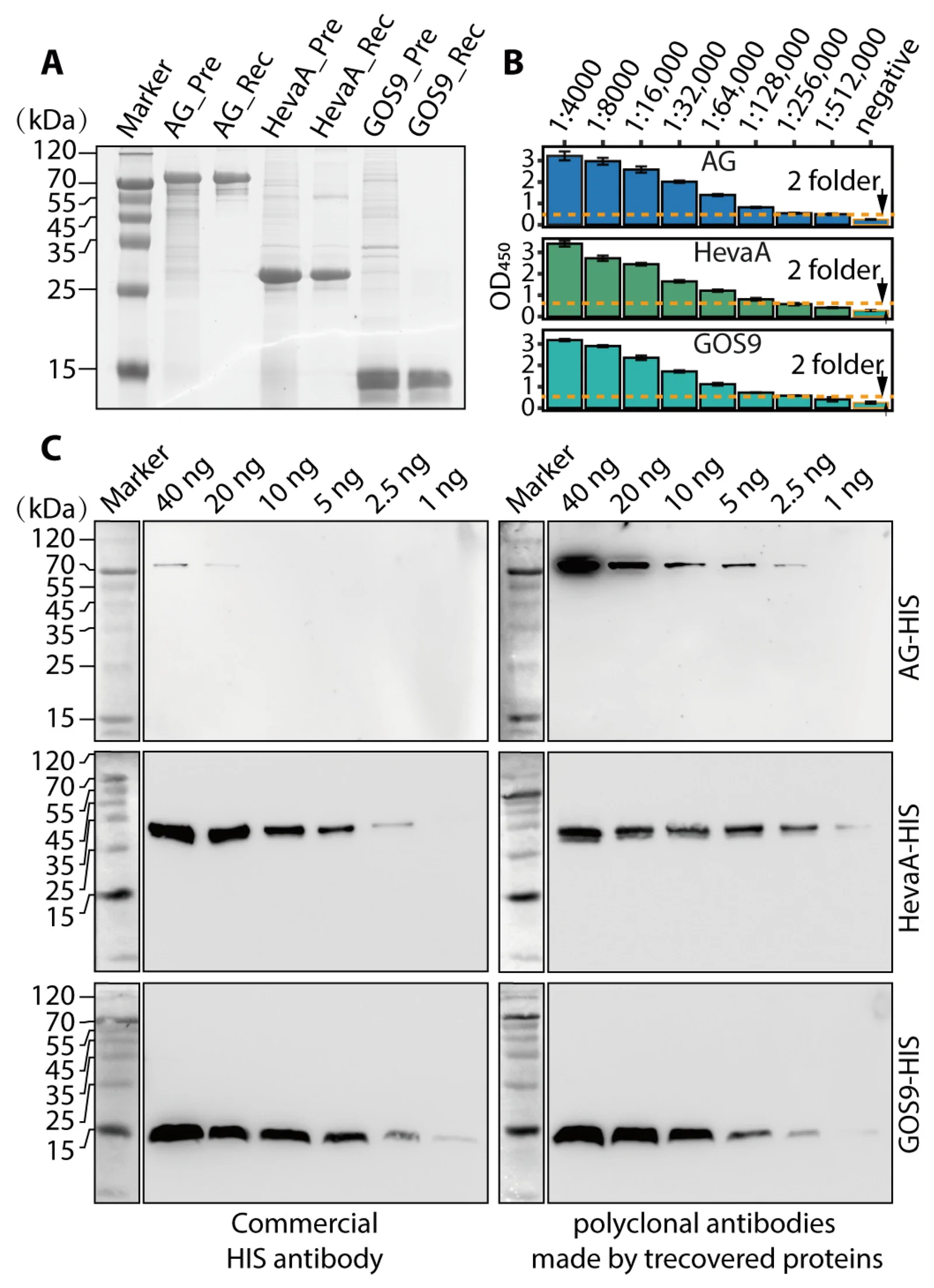
Recovery of prokaryotically expressed proteins for antibody production. (**A**) Purification of target proteins from *E. coli*. (**B**) Titer analysis of antibodies generated against recovered proteins. (**C**) Comparison of Western blot sensitivity between commercial His-tag antibodies and polyclonal antibodies produced from the recovered proteins. AG, α-galactosidase; HevaA, hevamine-A; GOS9, jacalin-like plant lectin. Pre, pre-recovery *E. coli* proteins; Rec, post-recovery proteins.

To evaluate whether antibody production via this newly developed method exhibits sensitivity comparable to that of commercial antibodies, we recovered prokaryotically expressed His-tagged proteins—α-galactosidase (AG), hevamine-A (HevaA), and jacalin-like plant lectin (GOS9)—from HAGs (Figure 5A) and generated corresponding polyclonal antibodies. All antibody titers exceeded 1:256,000 (Figure 5B). Under identical experimental conditions, Western blot analysis indicated that the sensitivity of the antibodies generated from the recovered proteins was either equivalent or superior to that of commercial His-tag antibodies (Figure 5C). Within our detection system, the AG protein showed reduced sensitivity, detectable at 2.5 ng, compared to HevaA and GOS9, which were detectable at 1 ng. This discrepancy is likely due to the lower transfer efficiency of HM_r_ proteins during blotting. Collectively, these results demonstrate that antigens purified using the newly established recovery method can be directly used for antibody production without requiring further purification.

We further applied the newly developed protein recovery method to purify high-abundance proteins from *Musa* leaves, pineapple pulp, and rubber tree latex. Electrophoresis analysis revealed that the purified samples from all three sources exhibited single bands (Figure S6A). Mass spectrometry analysis subsequently confirmed the predominant components within the purified fractions (Figure S6B). The recovered proteins, such as rubber elongation factor (REF) obtained from rubber tree latex, displayed high purity and could fully suitable for antibody preparation.

### 2.6. Molecular Mass-Based Fractionation for Proteomic Identification

**Figure 6:**
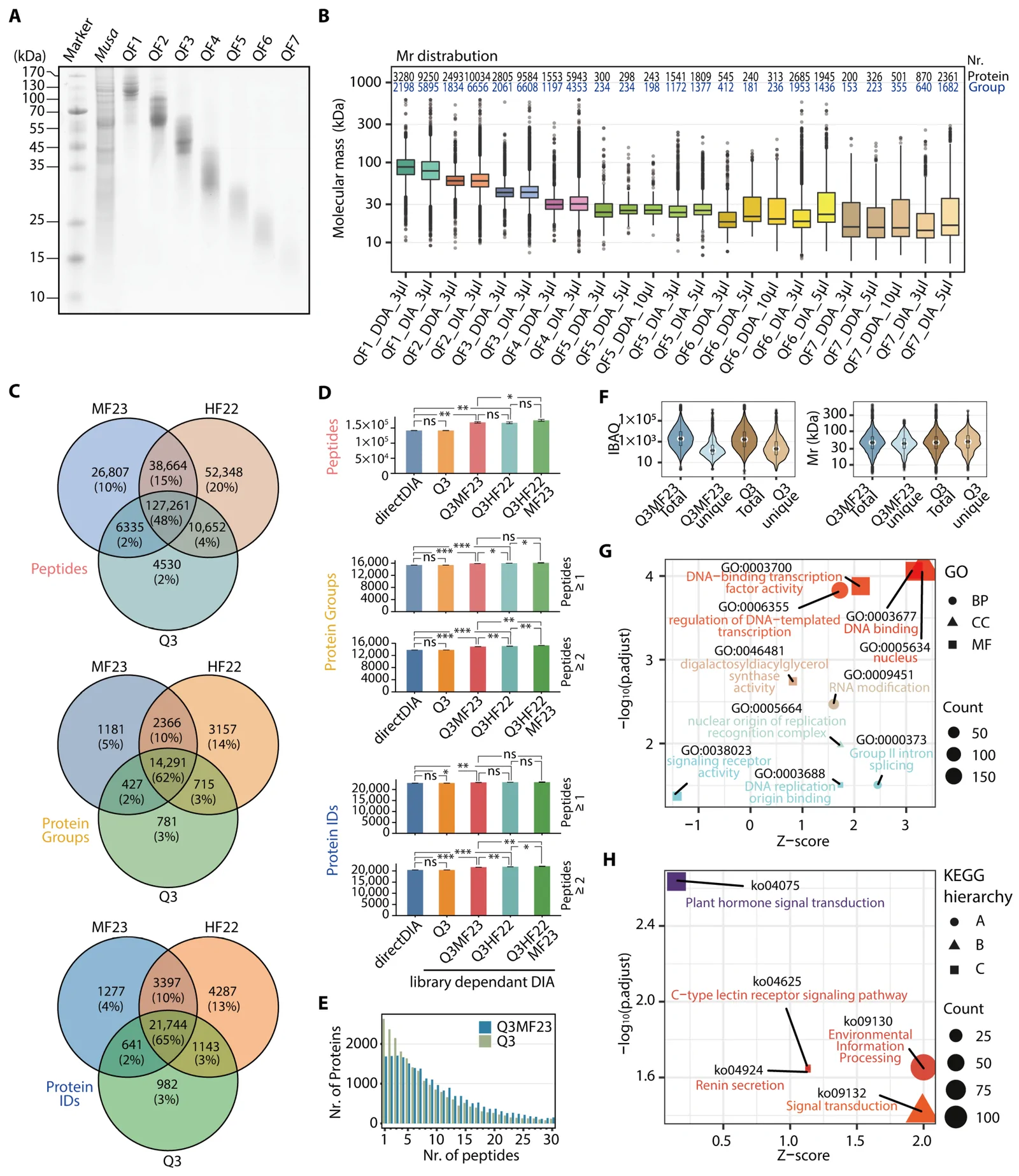
Proteome identification by directDIA and library-dependent DIA (based on molecular mass fractionation). (**A**) *Musa* protein fractions recovered from high-concentration agarose gels (HAGs). (**B**) Molecular mass (M_r_) distribution of protein fractions obtained via gel separation and silica column-based protein recovery. The numbers at the top of the figure indicate the numbers of identified proteins (Protein) and protein groups (Group) obtained under Data-Dependent Acquisition mode (DDA), Data-Independent Acquisition mode (DIA), and different injection volumes (3 μL, 5 μL, and 10 μL), for each fraction (QF1-7) respectively. (**C**) Library capacities generated from the recovered protein fractions (MF23), peptides fractions separated by high-pH reversed-phase liquid chromatography (HF22), and unfractionated samples (Q3). (**D**) Comparison of peptide and protein group yields between the directDIA and multiple library-dependent DIA approaches. *, *p* < 0.05, **, *p* < 0.01; ***, *p* < 0.001; ns, not significant. (**E**) Distribution of peptide counts matched to proteins identified using the Q3 library or the combined library Q3 and MF23 (Q3MF23). (**F**) Intensity-based absolute quantification (iBAQ) and M_r_ distributions of proteins uniquely identified in the comparison of Q3 and Q3MF23 library-dependent approach. (**G**) GO enrichment of proteins uniquely detected in the comparison of Q3 and Q3MF23 library-dependent approach. (**H**) KEGG enrichment of proteins uniquely detected in the comparison of Q3 and Q3MF23 library-dependent approach. QF, the pooled sample for library generation, created by combining equal portions of three *Musa* corm protein samples, followed by molecular mass-based fractionation and protein recovery; DDA, data-dependent acquisition; DIA, data-independent acquisition; directDIA, library-independent DIA; Z-score, represents the ratio of unique proteins from Q3MF23 library and Q3 library within the enriched categories or pathways.

Proteins extracted from *Musa* corm samples were separated by HAG electrophoresis (HAGE) and subsequently fractionated by M_r_ for recovery (Figure 6A). The seven resulting fractions were analyzed using both data-dependent acquisition (DDA) and data-independent acquisition (DIA) proteomics. These fractions were combined to construct a spectral library (MF23), with each fraction contributing from hundreds to thousands of protein groups (Figure 6B). The resulting comprehensive proteomic library comprised 199,067 peptides, 18,265 protein groups, and 27,059 proteins (Figure 6C). For comparison, a traditional library (HF22) constructed from peptide fractions separated by high-pH reverse-phase chromatography was also examined, which contained 228,925 peptides, 20,529 protein groups, and 30,571 proteins (Figure 6C). Both library construction methods yielded substantially higher identification numbers than the library built solely from the three unfractionated *Musa* corm samples (Q3), which contained 148,778 peptides, 16,214 protein groups, and 24,510 proteins, especially in terms of peptide identifications.

In subsequent proteomic analysis of *Musa* corm samples, supplementing the Q3 library with the MF23 library, the HF22 library, or a combination of MF23 and HF22 significantly increased the number of confidently identified peptides per sample. In contrast, although the numbers of identified protein groups and proteins also showed significant improvements, the increases were more modest (Figure 6D). Combining the MF23 library with the Q3 library enhanced the number of peptides identified per protein and substantially reduced the proportion of proteins identified by only 1–3 peptides (Figure 6E). When single peptide was used as the filtering threshold, the total number of proteins identified using the sample itself (via directDIA or the Q3 library) was 23,133 (with 1101 unique proteins compared to the combined Q3+MF23 library-based identification), whereas using the combined Q3+MF23 library yielded 23,483 total identified proteins (1451 unique). With a two-peptide filter, the sample-based approach identified 20,501 proteins (749 unique), compared to 21,799 proteins (2047 unique) using the Q3+MF23 library. The uniquely identified proteins were predominantly distributed in lower abundance regions, with those identified via the Q3+MF23 library occupying even lower abundance ranges, indicating that the use of MF23 library facilitated the detection of low-abundance proteins to some extent (Figure 6F).

GO enrichment analysis of the uniquely identified proteins revealed significant clustering in the “nucleus” for cellular component, “DNA binding” for molecular function, and “regulation of DNA-templated transcription” for biological process. The proportion of such proteins from using the Q3+MF23 library was much higher than from using Q3 library (Figure 6G). This suggests that improved identification of low-abundance proteins enhances the detection of proteins involved in signaling transduction-related functions, which is further corroborated by KEGG pathway enrichment in “Environmental information processing” and “Signal transduction” (Figure 6H). Together, these results demonstrate the potential feasibility of recovery-based protein fractionation for protein library generation in proteomic studies.

## 3. Discussion

The extraction and purification of plant proteins, especially target proteins, are often complex and labor-intensive processes. In contrast, nucleic acid purification offers significantly greater operational convenience. Mainstream nucleic acid purification methods using silica columns or silanol-functionalized magnetic beads primarily rely on electrostatic adsorption and hydrogen bonding between silanol groups and nucleic acids [19]. Since both protein and nucleic acids separated by gel electrophoresis carry negative charges, commercially available nucleic acid purification columns or magnetic beads could theoretically be adapted for rapid protein recovery. Previous studies have confirmed protein adsorption onto solid-phase resins following agarose gel dissolution with chaotropic salts [18]. Although agarose gels can be dissolved rapidly, an advantage over PAG for adsorption-based protein purification, PAG remains the conventional medium for protein separation. Historically, agarose gels were considered advantageous mainly for resolving high molecular mass (HM_r_) proteins (>200 kDa) [20].

Conventional protein separation requires high-concentration gels, and high concentration agarose gels (HAGs) used to be considerable challenges, limiting their utility in protein separation and related methodological development. However, our recent advances have enabled high-resolution protein separation via HAG electrophoresis (HAGE) [17], renewing our interest in gel-based protein recovery. In this study, we aimed to adapt the inexpensive, convenient, and widely adopted nucleic acid recovery workflow for rapid protein recovery, with the goal of developing a widely accessible method to facilitate protein purification.

Our results show that, among several common chaotropic salts tested for agarose gel dissolution, NaI yield the lowest protein flow-through (Figure 1A). Increasing the concentrations of NaI and NaCl in the binding buffer substantially enhanced protein adsorption onto silica columns (Figure 1B). This improvement can be attributed to Na^+^ ions neutralizing negative charges on both protein molecules and ionized silanol groups (Si-O^−^), thereby mitigating electrostatic repulsion, while I^−^ ions exert a dehydrating effect that promotes hydrogen bonding between Si-O^−^ and proteins. However, adsorption efficiency remained low for low-molecular-mass proteins (LM_r_, <25 kDa) under weakly alkaline or neutral conditions (Figure 1B,E). Previous studies indicate that adsorption of negatively charged molecules by silanol groups (Si-OH) is maximal at pH values near or below the pKa of the hydroxylated silica surface in high-ionic-strength buffers [19]. Isolated silanol groups have a pKa near 8.5, whereas those in cyclic silica oligomers exhibit pKa values around 4.5 [21]. Consistent with this, we improved the recovery of LM_r_ proteins by lowering the binding buffer pH below 5 (Figure 1F).

We observed that column-adsorbed proteins could be eluted directly with water prior to ethanol washing, similar to the elution behavior of nucleic acids (Figure 1C). However, after ethanol rinsing, efficient protein release required the presence of denaturants such as SDS or urea (Figure 1D and Figure S2B). Although ethanol was primarily used to remove salts and impurities, it likely also disrupts hydrophobic interactions between SDS and proteins, promoting complex dissociation and denaturation [22]. While ethanol-induced dehydration and precipitation of nucleic acids remain reversible, protein dehydration not only strips their hydration shell but also compromises internal hydrogen bonds and hydrophobic networks, leading to irreversible structural collapse [23,24]. This explains the requirement of denaturants for eluting proteins after ethanol washing. Native HAGE analysis further confirmed this. BSA and lysozyme eluted with an SDS-free lysis buffer showed electrophoretic mobility identical to that before recovery, whereas their migration patterns changed completely when SDS was included in the elution buffer (Figure S1), indicating effective removal of SDS during washing. This efficient stripping of denaturants might unlock more downstream applications, such as protein refolding.

Under non-reducing conditions, we observed minimal protein leakage in the binding buffer flow-through (Figure 1B,C), yet the final recovery yield was still compromised (Figure 1D). In contrast, adding reducing agents in the binding buffer markedly improved protein recovery (Figure 1E). These results suggest that the absence of reductants primarily impairs elution rather than initial adsorption. A plausible mechanism involves protein aggregation driven by covalent crosslinking and hydrophobic clustering [25], forming insoluble aggregates that resist conventional elution. Although cross-links like disulfide bonds are reductant-sensitive, the dense structure of such aggregates may sterically limit reductant access to buried sites during short elution steps. Moreover, non-reducible oxidative cross-links, such as dityrosine bonds and aldimine linkages, are even more irreversible [26,27]. Together, these factors likely cause adsorbed proteins to remain tightly bound to the silica column unless reducing agents are present at the beginning. Therefore, suppressing oxidation is critically important throughout recovery, especially during high-temperature gel dissolution. In addition to including reducing agents in the sol/binding buffer, future strategies could involve chelators like EDTA to further limit metal ion (e.g., Fe^3+^)-dependent oxidation [28], thereby enhancing recovery. Separately, we also found that dissolved gel solutions containing > 0.8% (*m*/*v*) agarose significantly lowered recovery efficiency (Figure S3), likely by impeding protein-silica membrane interaction. However, lower gel concentrations increased processing volume and column-passing steps, extending workflow duration and raising protein loss risk. Therefore, we determined an optimal agarose concentration of approximately 0.6% (*m*/*v*) (Figure S3), corresponding to a 1:14 volumetric ratio of HAG to sol/binding buffer (Figure 1G).

After establishing the method, we assessed its potential preferentiality by performing proteome recovery and mass spectrometry identification. The results show that the distributions of specifically expressed proteins (SEPs) and increasingly accumulated differentially expressed proteins (DEPs) in both *Arabidopsis* and *Musa* samples were significantly shifted toward LM_r_ range after recovery (Figure 3C). This finding contrasts with nucleic acid recovery, in which LM_r_ species are often recovered less efficiently, primarily due to adsorption limitations. We propose that for proteins, this difference may arise from the elution hindrance of HM_r_ proteins. Additionally, we also observed a weak but statistically significant negative correlation between protein abundance changes and isoelectric point (pI), but not with other physicochemical properties (Figure 3E). We hypothesized that the higher negative charge density of acidic proteins [29] might provide a competitive advantage in multi-protein environments. However, single-protein recovery assays showed no significant efficiency difference between acidic and basic proteins (Figure 4D), suggesting that charge-based preferential binding occurs primarily under competitive conditions. Furthermore, the single-protein recovery efficiency was comparable for HM_r_ and LM_r_ proteins (Figure 4D), supporting the broad applicability of the method across proteins with diverse properties.

At present, one primary application of the newly established protein recovery method is antigen preparation. Conventional approaches typically rely on recombinant expression of tagged target proteins, followed by affinity purification [30]. The efficiency of affinity purification, however, is often constrained by the expression state of the target proteins (soluble or inclusion bodies), tag accessibility, and nonspecific resin interactions, usually necessitating case-specific optimization of binding and elution conditions [4]. In contrast, our methods enable rapid, tag-free purification of target antigens directly from HAGs, irrespective of the protein’s physicochemical properties. The recovered antigens could be directly applied for antibody production and subsequent detection assays (Figure 5). Another promising application lies in proteomics. PAGE-based separation and purification have previously been utilized for MS identification of post-translationally modified isoforms [31,32], M_r_-based proteome pre-fractionation, and deep top-down proteomic analysis [15,33]. By integrating M_r_-based protein recovery into pre-fractionation for proteomics, we significantly enhanced proteomic identification depth and coverage (Figure 6). However, the current approach remains preliminary and exhibits several limitations. Separation resolution was suboptimal, largely due to gelatinous impurities in *Musa* corm extract, which impaired performance under high loading conditions (Figure 6A), and likely contributed to the substantial overlap observed between identified fractions (Figure 6B). Additionally, the use of an 8% (*m*/*v*) HAG resulted in uneven fractionation (Figure 6A), leading to a highly imbalanced distribution of identified proteins across fractions (Figure 6B) and possibly affecting overall proteome coverage. Future optimization could focus on improving sample purity and systematically evaluating lower gel concentrations (e.g., 6% (*m*/*v*) HAG) to achieve more balanced separation across M_r_ ranges and enhance the method’s utility in proteomic workflows.

## 4. Materials and Methods

### 4.1. Experimental Design

This study aimed to establish a convenient protein purification method. By adapting widely adopted nucleic acid purification workflows, this protein purification strategy is expected to achieve broader acceptance among users. The method employed high-resolution separation of proteins in high-concentration agarose gels (HAGs), in conjunction with commercially available silica columns originally designed for nucleic acid recovery. Through the development of optimized protein adsorption, rinsing, and elution buffer systems, efficient protein purification was accomplished. Subsequent validation experiments assessed the applicability of this approach in *E. coli* systems as well as in more complex plant protein purification. Large-scale recovery of plant proteomes and subsequent purification of distinct protein classes were performed to determine whether silica column-based purification exhibited selectivity based on protein properties. Further investigations evaluated the feasibility of using the purified proteins directly in downstream applications such as antibody production and proteomic analysis.

### 4.2. Acquisition of E. coli Proteins

*E. coli* BL21(DE3) (TransGen Biotech Co., Ltd., Beijing, China) cells were cultured overnight in Luria–Bertani (LB) medium until reaching an optical density (OD600) of approximately 4.0. The bacterial cultures were centrifuged at 13,680× *g* for 5 min to collect the cell pellets, which were then resuspended in 1/20 of the original culture volume of Tris-Cl buffer (50 mM, pH 6.8). The resulting suspension was transferred into 50 mL glass vessels placed in an ice-water bath and subjected to ultrasonic disruption using an ultrasonic homogenizer (Scientz Biotech, Ningbo, China) operating at 200 W for 5 min, with a cycle of 2 s on and 3 s off (all subsequent ultrasonic steps followed the same parameters). The lysate was clarified by centrifugation at 13,680× *g* for 5 min, and the supernatant was collected for protein quantification via the Bradford assay [34]. The total protein extract from BL21 (DE3) was stored at −80 °C for further use in establishing the protein purification methodology.

For the target protein genes—POBP5, Arg1B, ERP3, EIF1A, NOD93, LSM4, AG, HevaA, and GOS9—their open reading frames (ORFs) were cloned into the prokaryotic expression vector pET30a. These recombinant plasmids were transformed into Transetta (DE3) to generate corresponding recombinant strains. Each recombinant strain was cultured overnight in LB medium, after which 0.8 mL of the culture was inoculated into 80 mL of fresh LB medium and incubated at 37 °C with shaking at 180 rpm. When the OD600 reached approximately 0.8, protein expression was induced by adding IPTG to a final concentration of 0.5 mM, followed by continued incubation at 28 °C with shaking at 180 rpm for 5 h. Cells were harvested by centrifugation at 2380× *g* for 10 min. The cell pellets were resuspended in 8 mL of phosphate buffer (50 mM NaH_2_PO_4_, 300 mM NaCl, pH 8.0) and lysed by ultrasonic disruption. The lysates were then centrifuged at 13,680× *g* for 5 min at 4 °C. The insoluble fractions were collected, dissolved in 8 M urea, and stored at −80 °C for subsequent use in target protein recovery.

### 4.3. Extraction of Plant Proteins

The *Musa* plants (*Musa acuminata* AAA group ‘Brazilian’) were obtained from the Banana Tissue Culture Center, Institute of Banana and Plantains, Chinese Academy of Tropical Agricultural Sciences (Danzhou, Hainan, China). The plants were propagated in vitro from sucker tissues. Plant tissue (3 g) was cryogenically pulverized in liquid nitrogen with the addition of 0.1 g polyvinylpolypyrrolidone (PVPP) as a co-grinding agent. The resulting powder was transferred to a 50 mL centrifuge tube and homogenized with 10 mL of BPP extraction buffer (containing 100 mM ethylenediaminetetraacetic acid, 100 mM Tris-HCl, 50 mM borax, 50 mM vitamin C, 30% (*m*/*v*) sucrose, 2% (*v*/*v*) β-mercaptoethanol, 1% (*v*/*v*) Triton X-100, pH 8.0). The mixture was shaken at 2000 rpm for 10 min at room temperature. An equal volume of Tris-saturated phenol (pH ≥ 7.8) was then added, followed by an additional 10 min of shaking under the same conditions. The sample was centrifuged at 13,680× *g* for 15 min at 4 °C (conditions maintained for all subsequent centrifugation steps). The upper phenolic phase was carefully transferred to a new tube and combined with 5 volumes of saturated ammonium sulfate-methanol solution (AM) at 1.3% (*w*/*v*). Proteins were precipitated overnight at −20 °C, and the resulting precipitate was collected by centrifugation. The pellet was washed twice with pre-chilled methanol and acetone, with the supernatant removed after each wash step. The final pellet was air-dried and resuspended in 400 μL of lysis buffer (7 M urea, 2 M thiourea, 2% (*m*/*v*) CHAPS, 0.1 M PMSF, and 1% (*m*/*v*) DTT). Protein concentration was determined using the Bradford method [34], with bovine serum albumin (BSA) standards at concentrations ranging from 0 to 5 μg/μL. The isolated proteins were stored at −80 °C for subsequent analyses, including total proteome recovery and fractionated protein recovery.

### 4.4. Gel Separation of Proteins

HAGs were prepared in 1 M Tris-HCl (pH 8.8) according to a previously described method [17]. For comprehensive proteome recovery from *E. coli*, 5–6% (*m*/*v*) resolving gels were used, whereas for the entire plant proteome, 8% (*m*/*v*) resolving gels were employed. For the recovery of individual proteins, a 5% (*m*/*v*) stacking gel combined with 8–10% (*m*/*v*) resolving gels was utilized. In the M_r_-based fractionation of the *Musa* whole proteome, a 5% (*m*/*v*) stacking gel and an 8% (*m*/*v*) resolving gel were applied. Vertical HAG gels were cast in dimensions of 1 cm × 16 cm × 0.15 cm, and the horizontal HAG gels were cast in dimensions of 6.5 cm × 13 cm × 0.6 cm. All HAG separations were performed using vertical HAG electrophoresis (HAGE), except for two horizontal methods—native HAGE and standard horizontal HAGE—which were run at 100 V constant voltage in 200 mM Tris-HCl (pH 8.8). The vertical HAGE running conditions consisted of 200 mM Tris-HCl (pH 8.0) containing 0.1% (*m*/*v*) SDS as the cathode buffer and 200 mM Tris-HCl (pH 8.8) as the anode buffer, with electrophoresis carried out at a constant voltage of 200 V and a temperature of 16 °C. SDS-polyacrylamide gels (SDS-PAGE) were prepared and run according to the conventional protocol [35], using a discontinuous system comprising a 4% (*m*/*v*) stacking gel and a 12.5% (*m*/*v*) resolving gel, both prepared in Tris-Cl buffer (0.375 M Tris-Cl, pH 8.8, 0.1% (*m*/*v*) SDS), with an acrylamide-to-bisacrylamide ratio of 29:1. SDS-PAGE gels were cast in dimensions of 14 cm × 16 cm × 0.15 cm. Electrophoresis was performed in Tris-Gly buffer (containing 25 mM Tris, 200 mM glycine, 0.1% (*m*/*v*) SDS).

For protein detection in HAGs, three staining strategies were employed: a non-stained method for total protein recovery; chilled KCl (3 M, 4 °C, 10 min) for visualizing high-abundance target proteins; and Coomassie Brilliant Blue (CBB) staining [35] for detecting low- to moderate-abundance proteins and general visualization. For PAGs, proteins were visualized using a standard CBB staining protocol [35], followed by destaining in 10% (*v*/*v*) acetic acid. Gel images were acquired using a GE ImageScanner III (GE Healthcare, Chicago, IL, USA).

The concentrations of Tris-Cl used in the gel preparation and subsequent protein recovery are all indicated as the concentration of the Tris base component.

### 4.5. Protein Recovery

Proteins present in the flow-through from the binding and rinsing steps were concentrated using a 10 kDa molecular mass cut-off ultrafiltration device (Sartorius AG, Göttingen, Germany). For specific applications, silica columns were sourced as follows: *E. coli* whole proteome recovery used columns from the AxyPrep™ Plasmid Miniprep Kit (Corning Incorporated, Corning, NY, USA) and the Eastep™ Super Total RNA Extraction Kit (Promega Corp, Madison, WI, USA); method optimization and efficiency assessment used columns from the Eastep™ Super Total RNA Extraction Kit; antigen production and *Musa* proteome pre-fractionation used Abigen™ nucleic acid extraction columns (Abigen Biotech, Hangzhou, China). Protein samples, including *E. coli* lysate supernatants, flow-through fractions, and eluates, were analyzed by SDS-PAGE with equal-volume loading. Band intensity comparisons provided a visual assessment of recovery efficiency and procedural loss.

### 4.6. Analysis of Individual Protein Recovery Efficiency

To evaluate the recovery efficiency of individual proteins, standard samples of BSA and lysozyme, as well as purified expressed proteins, were subjected to HAGE at loading amounts of 5, 10, 20, 50, 100, and 200 μg. Following electrophoresis, the target protein bands were excised and recovered. Three independent experimental replicates were performed for each condition, with the exception of Arg1B, which was performed in duplicate. To avoid interference from sample buffer components and inaccuracies associated with traditional CBB staining or Bicinchoninic Acid (BCA) assays for low-concentration samples (<100 ng/μL), an image-based quantification approach was employed. Pre-recovery samples (0.625, 1.25, 2.5, 6.25, 12.5, and 25 μg) and post-recovery samples (1/8 of the recovery volume) were simultaneously separated by PAGE. After electrophoresis, gels were stained with CBB-G250 solution (12.5% (*m*/*v*) CBB-G250, 30% (*v*/*v*) ethanol, 10% (*v*/*v*) methanol, 6% (*v*/*v*) phosphoric acid). Gel images were analyzed using ImageMaster 2D Platinum software v5.0 (GE Healthcare, Chicago, IL, USA) to determine the volumetric percentage (Vol%) values of target protein bands in both pre- and post-recovery samples. A standard curve was generated based on the Vol% values of the pre-recovery samples. The recovery yield and recovery efficiency were subsequently calculated by applying the Vol% values of the post-recovery samples to this standard curve.

### 4.7. Antigen Purification and Antibody Preparation

The crude extract pellets of *E. coil* containing AG, HevaA, and GOS9 proteins were dissolved in 8 M urea and separated by HAGE. A total of approximately 2.4 mg of protein was loaded across two 14 cm wide HAGE gels. Following electrophoresis, the gels were stained with pre-chilled 3 M KCl at 4 °C for 10 min, and distinct white protein bands were excised for recovery. The target proteins were then purified using twelve 2 mL silica columns. The eluted proteins were quantified and directly used as antigens for rabbit immunization. For the primary immunization, 800 μg of the target protein emulsified in Freund‘s complete adjuvant was administered subcutaneously. Two weeks later, three booster injections were performed at weekly intervals, each containing 300 μg of target proteins mixed with Freund’s incomplete adjuvant. Serum was collected one week after the final immunization for subsequent antibody titer analysis and Western blot validation.

### 4.8. Titer Analysis

Antibody titers were determined using a rabbit polyclonal antibody titer assay kit (RuiXin Biotech, Wuhan, China), with serial dilutions of the primary antibody assessed for chromogenic signal. Reactions without the primary antibody served as the background control. Briefly, the antigen-coated plate was incubated overnight at room temperature with the antigen dilution buffer (supplied within the kit). The following day, the coated antigen was blocked with kit-provided blocking buffer for 2 h at 37 °C. After removal of the blocking solution, the plate was air-dried at room temperature for 6 h. Serial dilutions of the primary antibody (ranging from 1:4000 to 1:512,000) were applied to the wells. The plate was sealed and incubated at 37 °C in the dark for 30 min. Following incubation, the liquid was discarded, and the plate was washed five times with the provided wash buffer. HRP-conjugated secondary antibody (included in the kit) was then added, and the plate was resealed and incubated again at 37 °C in the dark for 30 min. After another five washes, substrate solution was added, and the plate was resealed and incubated under the same conditions for 15 min. The reaction was terminated by adding the stop solution, and the absorbance at 450 nm (OD450) was immediately measured using an Epoch microplate reader (BioTek, Shoreline, WA, USA).

### 4.9. Western Blot

For Western blot (WB) analysis, proteins separated by SDS-PAGE were transferred to PVDF membranes using a semi-dry transfer system (Beijing Liuyi Biotech, Beijing, China). The transfer buffer consisted of 25 mM Tris, 192 mM glycine, and 20% (*v*/*v*) methanol, and transfer was conducted at a constant current of 1.5 mA/cm^2^ for 2 h. The PVDF membrane was subsequently blocked with 5% (*m*/*v*) skim milk in TBST (20 mM Tris, 150 mM NaCl, 0.05% (*v*/*v*) Tween 20, pH 7.4) under gentle agitation (50 rpm) for 1.5 h at room temperature. The membrane was then incubated with primary antibodies diluted 1:1000 in the same blocking buffer: either a His tag mouse monoclonal antibody (Shanghai Beyotime Biotech, Shanghai, China) or custom-made polyclonal antibodies (rabbit) raised against the target proteins, with continued gentle shaking for 1.5 h. Thereafter, a horseradish peroxidase (HRP)-conjugated secondary antibody (goat anti-mouse or goat anti-rabbit; Shanghai Beyotime Biotech, Shanghai, China) was applied at a dilution of 1:10,000 in TBST containing 5% (*m*/*v*) skim milk, and the membrane was incubated under gentle agitation for 1 h at room temperature. After thorough washing, protein bands were visualized using BeyoECL SuperMoon (Shanghai Beyotime Biotech, Shanghai, China), and chemiluminescent signals were immediately captured with an LAS4000mini imaging system (GE Healthcare, Chicago, IL, USA).

### 4.10. Proteomic Pre-Fractionation

For M_r_-based fractionation, three protein samples extracted from individual *Musa* corms (~300 μg each) were pooled together and separated on a discontinuous HAG system consisting of a 5% (*m*/*v*) stacking gel and an 8% (*m*/*v*) resolving gel. After electrophoresis, performed without staining, the gel lane was systematically excised into seven sequential segments from the well to the dye front, corresponding to predefined molecular mass ranges. Proteins from each excised segment were recovered using the silica gel column-based purification. The resulting fractions were served as pre-fractions for downstream digestion and proteomic library construction. For traditional fractionation, the pooled *Musa* corms samples (~50 μg each) were digested by Trypsin/Lys-C first, then separated by high-pH reverse-phase (HpH) chromatography, and combined to eleven fractions.

### 4.11. Trypsin/Lys-C Digestion

For protein fractions obtained from recovery and the unfractionated protein samples planned to identification, 5–20 μg of protein were reduced with 1.25–5 μL 1 M DTT (dissolved in 8 M urea) at 37 °C for 1 h first. Subsequently, alkylation was performed by adding 5–20 μL of 1 M iodoacetamide (dissolved in 8 M urea) followed by incubation in the dark at room temperature for 1 h. The reaction mixture was then transferred to a 10 kDa molecular mass cut-off ultrafiltration tube and centrifuged at 13,680× *g* for 20 min and washed twice with 8 M urea. For enzymatic digestion, 6.25–25 μL of digestion buffer (50 mM ammonium bicarbonate, pH 8.0) containing 0.5–2 μg of Trypsin/Lys-C (Promega Corp, Madison, WI, USA) was added to the ultrafiltration unit, and the mixture was incubated at 37 °C for 16 h. For proteins used for HpH fractionated, ~150 μg of proteins were executed trypsin/Lys-C digestion. The resulting peptides were collected by centrifugation and lyophilized for subsequent liquid chromatography-tandem mass spectrometry (LC-MS/MS) analysis. All reagents used in this study are listed in Table S3.

### 4.12. MS Data Acquisition and Processing

The mass spectrometry data were acquired in a single batch on an Orbitrap Exploris 480 mass spectrometer (Thermo Fisher Scientific, Waltham, MA, USA), using data-independent acquisition (DIA) mode with a 1 h gradient. Raw files were analyzed with Spectronaut (v18) against the *Musa* proteome database (NCBI accession GCF_000313855.2). The data analysis was acquired on an Orbitrap Astral Zoom system, operating in either data-dependent acquisition (DDA) or DIA mode (1 h gradient). While fractionated samples were measured in both modes, the three unfractionated *Musa* corm samples were analyzed using DIA only.

Three spectral libraries were constructed from these data using Spectronaut (v19) and the same protein database, with a false discovery rate (FDR) threshold of ≤1%. The Q3 library was generated from the three *Musa* corm DIA runs. The MF23 library was derived from thirteen DDA and ten DIA runs of seven M_r_-separation-based fractions. The HF22 library was built from eleven DDA and eleven DIA runs of peptide fractions separated by high-pH reverse-phase chromatography. Subsequently, the three *Musa* corm DIA datasets were analyzed via directDIA and library-based searches using the Q3, Q3+MF23, Q3+HF22, and combined Q3+MF23+HF22 libraries, all with default software parameters.

### 4.13. Data Analysis

Protein M_r_, grand average of hydropathicity (GRAVY), and isoelectric point (pI) were calculated using the BioPython package (v1.81) in Python (v3.9.13). Statistical distribution fitting was performed in R (v4.0.2). Protein abundance, M_r_, and GRAVY were fitted to a normal distribution by estimating the mean (μ) and standard deviation (σ), with probability density calculated using the built-in dnorm() function. For bimodally distributed pI data, a two-component Gaussian mixture model (GMM) was fitted via the normalmixEM() function from the mixtools package (v1.2.0) using the Expectation-Maximization algorithm, estimating for each component the μ, σ, and mixing proportion (λ).

Between-group differences were evaluated using two-tailed Student’s *t*-tests, with a nominal significance threshold of *p* ≤ 0.05. *p*-values derived from proteomic comparisons were further adjusted using the Benjamini–Hochberg (BH) method to generate q-values; q ≤ 0.05 was considered statistically significant. The relationships between accumulation changes in protein (ratio between groups) and protein properties, mean abundance (PG.Quantity), M_r_, pI, and GRAVY, were evaluated using non-parametric Spearman’s rank correlation analysis in R. Spearman’s ρ and *p*-values were calculated with the cor.test() function. Correlations meeting both criteria of 0.1 ≤ |ρ| < 0.4 and *p* < 0.05 were defined as statistically significant and weakly correlated [36].

For Gene Ontology (GO) and Kyoto Encyclopedia of Genes and Genomes (KEGG) enrichment analyses, protein identifiers were annotated with GO terms using the UniProt ID-mapping tool (accessed 16 January 2026) and assigned KEGG Orthology (K) identifiers via BlastKOALA (15 January 2026) [37]. Detailed category and hierarchy information was retrieved from the go.obo (release 16 March 2025) and ko00001.keg (downloaded 14 January 2025) files, yielding comprehensive annotation files for proteins identified with the Q3 and Q3+MF23 spectral libraries. Enrichment analysis was conducted using the clusterProfiler package (v4.16.0) in R (v4.5.1) [38].

## 5. Conclusions

We have established a silica-column-based method that makes protein recovery from gels as simple and rapid as nucleic acid purification, requiring only routine centrifugation and no specialized equipment. This workflow bridges gel electrophoresis with downstream proteomic applications, enabling efficient antigen purification and proteomic DIA library construction. Although the current reliance on in-house gel preparation and commercially available columns introduces some practical variability, the overall accessibility and scalability of the method are expected to facilitate its broad adoption. Future commercial development of pre-cast gels and protein-optimized columns would further enhance its utility in a wider range of protein research and applications.

## Data Availability

All data needed to evaluate the conclusions in the paper are present in the paper or the Supplementary Materials. The proteomic data generated in this study have been deposited to the ProteomeXchange Consortium via PRIDE under identifiers PXD074029 and PXD074056.

## Supplementary Materials

The following supporting information can be downloaded at https://www.mdpi.com/article/10.3390/molecules31183307/s1, Figure S1: Native HAG electrophoresis (HAGE) of recovered BSA and LYS proteins by different elution buffers; Figure S2: Effect of β-Mercaptoethanol in binding buffer on protein recovery; Figure S3: Impact of agarose concentration on protein recovery; Figure S4: Recovered samples of the whole proteome of *Arabidopsis* and *Musa*; Figure S5: Fitted distribution curves of different protein types across various protein properties pre- and post-recovery; Figure S6: Recovery of high-abundance proteins from plant tissues; Table S1: Specifications and manufacturer/supplier of the silica columns used in Figure 2A; Table S2: Fitting curve parameters for proteins abundance, M_r_, hydro and pI between pre- and post-recovery protein samples in Figure S5; Table S3: Information for reagents used in the study.

## Abbreviations

The following abbreviations are used in this manuscript:

2D-PAGE	two-dimensional polyacrylamide gel electrophoresis
2ME	β-mercaptoethanol
AG	α-galactosidase
AM	Saturated ammonium sulfate-methanol solution
Arg1B	Argonaute 1B-like
BCA	Bicinchoninic acid
BPP	The protein extraction buffer
BSA	Bovine serum albumin
CBB	Coomassie brilliant blue
CI	Confidence interval
DDA	Data-dependent acquisition
DEPs	Differentially expressed proteins
DIA	Data-independent acquisition
DTT	DL-dithiothreitol
ERP3	Elicitor-responsive protein 3-like
ETIF1A	Eukaryotic translation initiation factor 1A-like
FPLC	Fast protein liquid chromatography
GITC	Guanidine thiocyanate
GMM	Gaussian mixture model
GRAVY	Grand average of hydropathicity
GuHCl	Guanidine hydrochloride
HAG	High-concentration agarose gel
HAGE	HAG electrophoresis
HevaA	Hevamine-A
HM_r_	High molecular-mass
iBAQ	Intensity-based absolute quantification
LB	Lysis buffer
LC-MS/MS	Liquid chromatography-tandem mass spectrometry
LM_r_	Low molecular-mass
LSm4	Probable U6 snRNA-associated Sm-like protein LSm4
LYS	Lysozyme
MALDI-MS	Matrix-assisted laser desorption-ionization mass spectrometry
MS	Mass spectrometry
NOD93	Early nodulin-93-like
ORFs	Open reading frames
PAG	Polyacrylamide gels
POBP5	Peroxisome biogenesis protein 5
PVPP	Polyvinylpolypyrrolidone
REF	Rubber elongation factor
SEPs	Specifically expressed proteins
