# Supplementary material for: A Novel Protein Recovery Method via Gel Separation and Silica Columns Capable of Rapid Antigen Purification and Deep Proteomic Identification
Source: Molecules. 2026 Sep 17;31(18):3307. doi: 10.3390/molecules31183307 (PMC13609993; doi:10.3390/molecules31183307)
Supplement: Supplementary file 1 [file molecules-31-03307-s001.zip › molecules-4501105-supplementary.pdf]

Supplementary Materials

This PDF file includes:

Figure. S1 to S6
Table S1 to S3

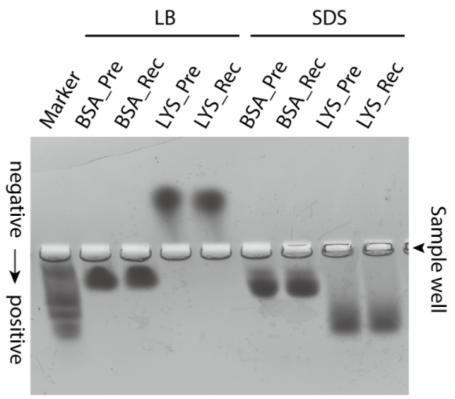

Figure S1. Native HAG electrophoresis (HAGE) of recovered BSA and LYS proteins by different elution buffers. LB, proteins eluted with lysis buffer loaded using SDS-free loading buffer; SDS, the elution buffer eluted proteins loaded with SDS-containing loading buffer. BSA, bovine serum albumin; LYS, lysozyme. Pre, pre-recovery protein standards; Rec, post-recovery proteins. Electrophoresis was performed using a 5% horizontal HAG; neither the gel buffer (1 M Tris-Cl, pH 8.8) nor the running buffer (0.2 M Tris-Cl, pH 8.8) contained SDS.

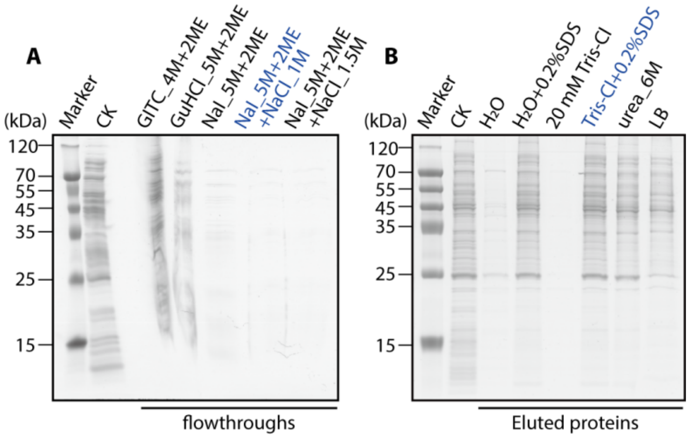

Figure S2. Effect of β-Mercaptoethanol in binding buffer on protein recovery. (A) Analysis of protein flow-through under different binding buffers containing 1% β-mercaptoethanol (2ME). (B) Protein recovery efficiency of various elution buffers following the use of a binding buffer composed of 5 M NaI, 1 M NaCl, and 1% 2ME. CK, 25 µg of *E. coli* protein (pre-recovery); recovered proteins (from 200 µg of *E. coli* protein) were loaded at 1/8 of the eluent volume; GITC, guanidine isothiocyanate; GuHCl, guanidine hydrochloride; LB, lysis buffer (7 M urea, 2 M thiourea, 4% CHAPS, 2% DTT, pH 8.5).

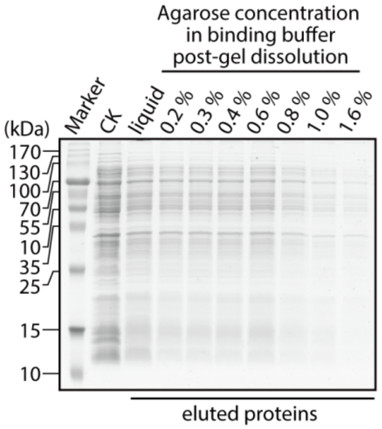

Figure S3. Impact of agarose concentration on protein recovery. CK, 25 µg of *E. coli* proteins prior to recovery; liquid, direct recovery of *E. coli* proteins without gel separation; Proteins recovered from 200 µg of *E. coli* protein were loaded at 1/8 of the eluent volume.

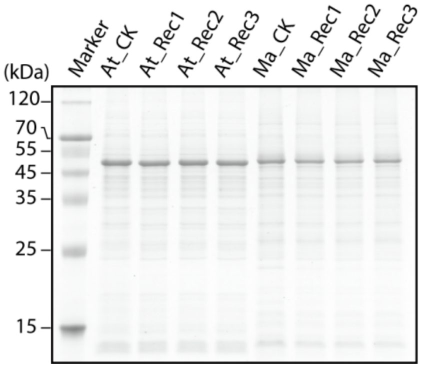

Figure S4. Recovered samples of the whole proteome of *Arabidopsis* and *Musa*. Proteins were recovered from 200 µg of *Arabidopsis* (At) or banana (Ma) extracts, with each sample loaded at 1/8 of the recovered volume. CK, 25 µg proteins pre-recovery; Rec1-3, three independent recovery replicates.

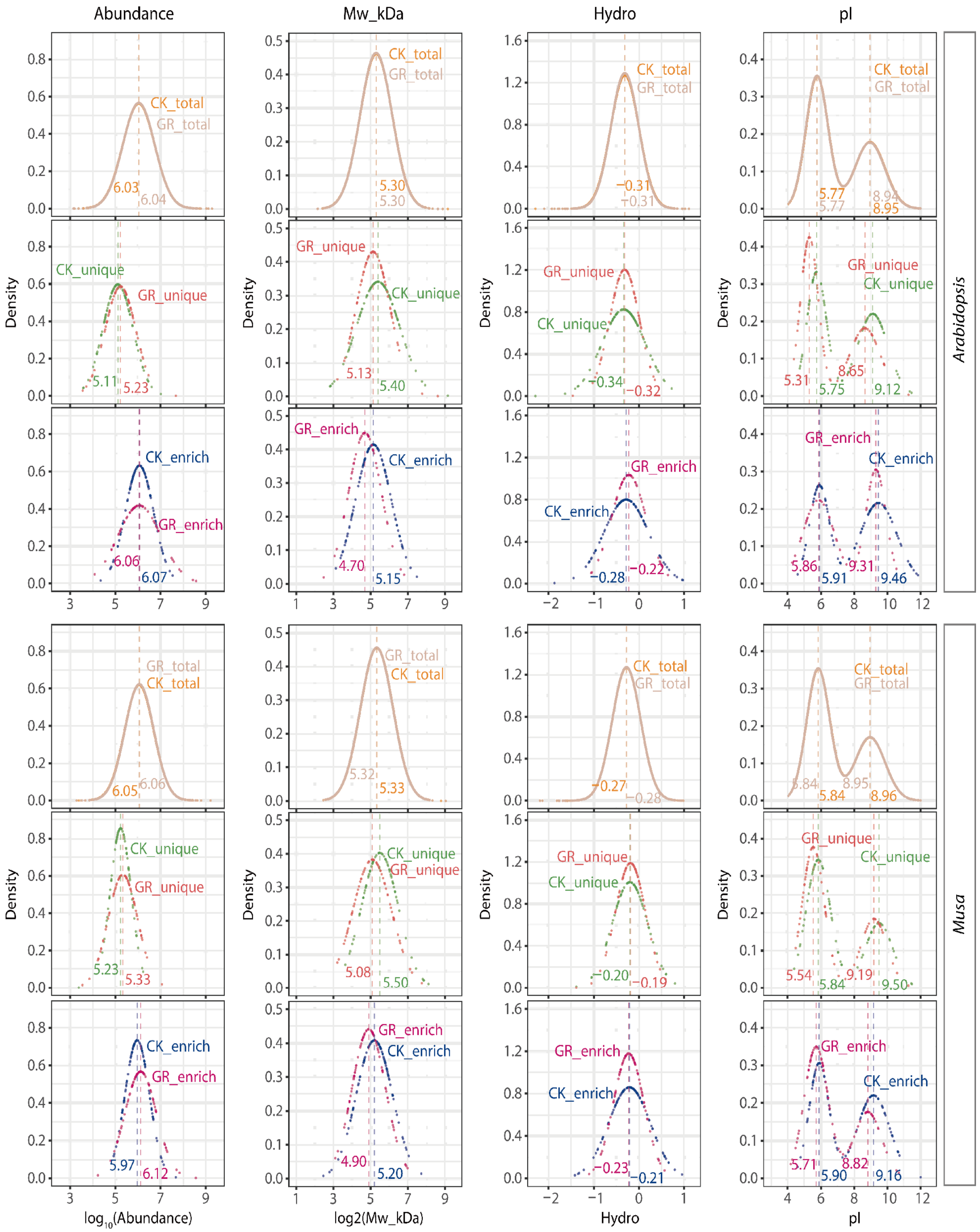

Figure S5. Fitted distribution curves of different protein types across various protein properties pre- and post-recovery. CK_total, all identified proteins pre-recovery; GR_total, all identified proteins post-recovery; CK_unique, proteins specifically expressed only pre-recovery; GR_unique, proteins specifically expressed only post-recovery; CK_enrich, differentially expressed proteins enriched in samples pre-recovery; GR_enrich, differentially expressed proteins enriched in samples post-recovery.

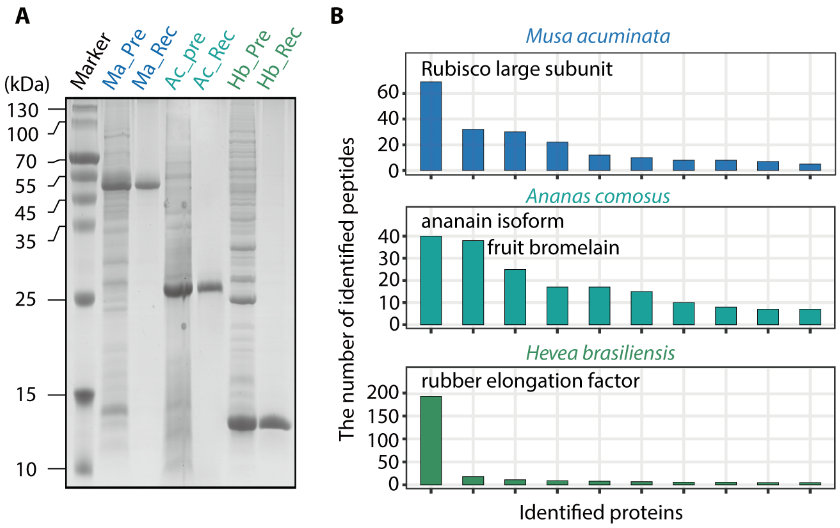

Figure S6. Recovery of high-abundance proteins from plant tissues. (A) Target proteins recovered from banana leaves (Ma), pineapple pulp (Ac), and rubber tree latex (Hb). (B) Mass spectrometric identification of the recovered proteins. Pre, pre-recovery proteins; Rec, post-recovery proteins.

Table S1. Specifications and manufacturer/supplier of the silica columns used in Figure 2A.

Lane	Specifications	Manufacturer/Supplier
AP-MN-P_Plasmid	AxyPrep™ Plasmid Miniprep Kit	Corning Incorporated, New York, USA
DP107_Plasmid	High-purity plasmid mini-preparation kit	Tiangen Biotech, Beijing, China
BW-DC3511_PCR	DNA Gel/PCR Purification Miniprep Kit	Beiwo Medical, Zhejiang, China
GN_2303_PCR	PCR Product DNA Purification Kit	GENENODE™, Wuhan, China
TR_9762_Gel	MiniBEST Agarose Gel DNA Extraction Kit	TAKARA Bio, Kusatsu, Japan
AP-GX_Gel	AxyPrep™ DNA Gel Extraction Kit	Corning Incorporated, New York, USA
D3187-01_DNA	RaPure Plant DNA Kit	Magen Biotech, Guangzhou, China
D3390-01_DNA	E.Z.N.A.™ Fungal DNA Kit	Omega Biotek, Georgia, USA
LS1040_RNA	Eastep™ Super Total RNA Extraction Kit	Promega Corp, Wisconsin, USA
DP419_RNA	RNA simple Total RNA Kit	Tiangen Biotech, Beijing, China
R6842_miRNA	E.Z.N.A.™ miRNA Isolation Kit	Omega Biotek, Georgia, USA

Table S2. Fitting curve parameters for proteins abundance, M_r, hydro and pI between pre- and post-recovery protein samples in Figure S5.

Class	Abundance_mean	Mw_mean	Hydro_mean	pI_mu1	pI_mu2	pI_sigma1	pI_sigma2	pI_lambda1	pI_lambda2	Species
CK_total	6.035	5.297	-0.312	5.774	8.951	0.667	0.911	0.593	0.407	*Arabidopsis*
GR_total	6.038	5.295	-0.312	5.766	8.941	0.668	0.909	0.595	0.405	*Arabidopsis*
CK_unique	5.112	5.398	-0.337	5.749	9.117	0.589	0.922	0.490	0.510	*Arabidopsis*
GR_unique	5.226	5.132	-0.318	5.309	8.649	0.541	0.931	0.576	0.424	*Arabidopsis*
CK_enrich	6.065	5.146	-0.280	5.912	9.463	0.602	1.117	0.396	0.604	*Arabidopsis*
GR_enrich	6.062	4.696	-0.224	5.859	9.307	0.951	0.610	0.534	0.466	*Arabidopsis*
CK_total	6.054	5.330	-0.275	5.843	8.958	0.697	0.902	0.617	0.383	*Musa*
GR_total	6.060	5.325	-0.275	5.838	8.954	0.695	0.901	0.616	0.384	*Musa*
CK_unique	5.233	5.496	-0.198	5.845	9.496	0.740	0.835	0.637	0.363	*Musa*
GR_unique	5.331	5.084	-0.187	5.542	9.190	0.666	0.797	0.630	0.370	*Musa*
CK_enrich	5.967	5.200	-0.211	5.897	9.165	0.602	0.974	0.461	0.539	*Musa*
GR_enrich	6.121	4.902	-0.226	5.714	8.825	0.721	0.833	0.632	0.368	*Musa*

Table S3. Information for reagents used in the study.

Name of the relevant reagents	Product code No.	Factory
Agarose	A620014-0100	Sangon Biotech, China
2-carboxyethyl)phosphine hydrochloride hydrochloride (Tris)	T8230-500g	Beijing Solarbio Science & TeChinaology, China
hydrochloric acid (HCl)	CB21-CP-01KG	Guangzhou Chemical, China
Sodium dodecylsulfate (SDS)	S8013-100g	Beijing Solarbio Science & TeChinaology, China
Acrylamide	A601032-0250	Sangon Biotech, China
Bis-acrylamide	A600025	Sangon Biotech, China
Ammonium persulfate (APS)	A600072-0025	Sangon Biotech, China
N,N,N',N'-Tetramethylethylenediamine (TEMED)	A610508	Sangon Biotech, China
Glycine	G8200-500g	Beijing Solarbio Science & TeChinaology, China
Potassium chloride (KCl)	AC06-GR-500G	Guangzhou Chemical, China
Coomassie brilliant blue G-250	A600038-0025	Sangon Biotech, China
acetic acid	CB39-AR-0.5L	Guangzhou Chemical, China
Sodium iodide (NaI)	BC07-AR-500G	Guangzhou Chemical, China
Sodium chloride (NaCl)	BC13-AR-500G	Guangzhou Chemical, China
2-Mercaptoethanol	A355395	Sangon Biotech, China
Ethanol absolute	HB15-AR-0.5L	Guangzhou Chemical, China
Trypsin	VA9000	Promega Corp, USA
Yeast extract	LP0021B	Thermo Scientific™ Oxoid, Britain
agar	ST004E	Shanghai Beyotime Biotech, China
Poly-vinylpolypyrrolidone	A500752-0050	Sangon Biotech, China
Sodium tetraborate pentahydrate	BG12-AR-500G	Guangzhou Chemical, China
Ethylenediaminetetraacetic acid disodium salt	BH01-AR-500G	Guangzhou Chemical, China
Sucrose	IB11-AR-500G	Guangzhou Chemical, China
L-Ascorbic acid	A100143-0100	Sangon Biotech, China
TritonX-100	TritonX-100	Sigma-Aldrich, USA
Tris-saturated phenol	SL2020-250mL	Coolaber, China
Ammonium sulfate	DC08-AR-500G	Guangzhou Chemical, China

Methanol	HB05-AR-0.5L	Guangzhou Chemical, China
Acetone	HB02-AR-0.5L	Guangzhou Chemical, China
urea	33247-250G	Sigma-Aldrich, USA
thiourea	T7875-100G	Sigma-Aldrich, USA
CHAPS	20102ES03	YEASEN, China
PMSF	P0100-10ml	Beijing Solarbio Science & TeChinaology, China
DTT	ID5851	Beijing Solarbio Science & TeChinaology, China
BSA	P0007	Shanghai Beyotime Biotech, China
Sodium phosphate monobasic (NaH_2PO_4)	BG05-AR-500G	Guangzhou Chemical, China
Freund's complete adjuvant	P2036-50ml	Shanghai Beyotime Biotech, China
Freund's incomplete adjuvant	P2031-50ml	Shanghai Beyotime Biotech, China
Rabbit polyclonal antibody titer assay kit (ELISA)	RXTY024	RUIXIN, China
PVDF membrane	10600023	GE Healthcare, USA
Skim milk	P0216-300g	Shanghai Beyotime Biotech, China
Tween-20	A100777-0500	Sangon Biotech, China
HRP-conjugated goat anti-mouse secondary antibodies	A0354	Shanghai Beyotime Biotech, China
BeyoECL Star Chemiluminescence Kit	P0018AM	Shanghai Beyotime Biotech, China
NH4HCO3	A6141-25G	Sigma-Aldrich, USA
Iodoacetamide (IAA)	A600539-0025	Sangon Biotech, China
Trifluoroacetic acid	302031-100ML	Sigma-Aldrich, USA
Acetonitrile	900667-1L	Sigma-Aldrich, USA
Formic Acid	186006691	Waters Corporation, USA
